# A Framework for Ethical and Clinical Governance Evaluation of Innovative Medicines in Children: A Mixed Methods Study

**DOI:** 10.1111/jpc.70501

**Published:** 2026-07-25

**Authors:** Sonya Stacey, Jamie Lam, Fiona Gregory, Shakira Wild, Steven McTaggart, Helen Irving

**Affiliations:** ^1^ Queensland Children's Hospital South Brisbane Australia; ^2^ The University of Queensland St Lucia Australia

## Abstract

**Aim:**

The aim of this project was to develop and implement a working definition and ethical framework for use of innovative medicine in contemporary Australian paediatric practice.

**Methods:**

A mixed methods research methodology was undertaken using a Delphi process to establish a definition of innovative medicines in paediatric patients. This definition was used to quantitate innovative medicines use in a retrospective quantitative review of non‐formulary medications in a tertiary paediatric hospital. An existing ethical framework from the United Kingdom was adapted to supplement standard clinical governance processes in contemporary Australian paediatric practice.

**Results:**

A definition of innovative medicines and an ethical framework for their use in paediatric patients was developed after four rounds of feedback from 18 Australian paediatric experts. A total of 2906 individual patient medication approval applications were reviewed, 1447 of these were included for analysis, 120 of which were for medications not on the Australian Register of Therapeutic Goods and six applications were considered innovative using the newly developed definition.

**Conclusions:**

Evaluation of the use of innovative medicines in paediatric patients is essential to ensure patient safety and critical clinical governance of medication use. An adapted ethical framework for Australian practice is a valuable tool to assist clinical governance processes.

## Brief Points

- What is already known on this topic
  ○ Clinical ethics consultations are increasingly used in paediatric practice, including in decision making regarding possible use of innovative therapies.
  ○ An ethical framework developed in the United Kingdom has been used in some Australian paediatric settings.
- What this paper adds
  ○ An adapted ethical framework to supplement clinical governance processes for the oversight and use of innovative medicines in paediatric patients appropriate for the Australian context.

## 1 Introduction

The rapid advancement of new and innovative therapies to treat rare or otherwise untreatable conditions in paediatric patients results in clinical, financial and ethical dilemmas for healthcare providers caring for young persons and families. The escalating complexity and cost of new therapies, particularly medicines, along with increasing complexity of medicines access schemes, necessitates appropriate governance and ethical consideration over clinical use of new and novel medicines, particularly in paediatric patients [1].

Paediatric healthcare institutions across the world face the common challenge of frequent use of off‐label and unlicensed medicines in paediatric patients [2, 3, 4]. This creates medication governance challenges that require robust assessment processes, especially in the treatment of rare conditions with novel therapies. Assessing the safety of innovative medicines based upon limited evidence, while balancing the need for equity of access to often extremely high cost medicines, is critical in oversight of these therapies [5, 6, 7]. Within the Australian healthcare context, differing local governance systems to manage these concerns can result in duplication of effort and gaps in equity across the country [8, 9]. Although substantial efforts have been made in recent years to standardise approaches to decision‐making and to share learnings regarding novel and high‐cost therapies across Australian paediatric centres [10] further work is needed to establish a clear and practical definition of what constitutes an innovative medication therapy in paediatric patients [8, 9, 10]. Such a definition would enable consistent identification of therapies that warrant additional operational and ethical consideration and evaluation.

The current approach by most Australian paediatric hospitals for approval of medication use outside of standard formulary restrictions involves an individual patient approval (IPA) process (also referred to as individual patient use or IPU) which provides governance over prescribing outside of the limits set in the formulary. Novel or costly therapies will often be escalated to a Medicines and Therapeutics Committee (MTC) for consideration. Within Australia, guiding principles for the conduct of these committees are provided by the Council of Australian Therapeutic Advisory Groups [11], with the aim to promote inter‐jurisdictional consistency in approach to medicines governance. While these guiding principles include the need to implement ethically robust processes, the focus is on equity of access, conflicts of interest, resource sustainability and confidentiality and little detail is provided on the processes underpinning ethical assessment.

Within our setting, the Medicines Advisory Committee (MAC) has responsibility for managing the overarching governance for medicines use. While this committee routinely assesses IPU/IPA applications, there was previously no standardised process to discuss ethical issues in relation to non‐formulary medication use. On occasion, these concerns may have been considered by the organisation's Clinical Ethics Consultation Service (CECS), which discusses cases where ethical concerns within or between clinical teams or with the family are deliberated, using a process described by Jansen et al. [12]. In cases involving novel medicines, CECS utilised an ethical framework including eight guiding principles for evaluation of innovative therapies in paediatric patients that was developed at the London Great Ormond Street Hospital for Children (GOSH) in 2009 by Brierley et al. [1]. They defined innovative therapy as *‘*any newly introduced treatment, or a new modification to an existing therapy with unproven efficacy and side effect profile, which is being used in the best interests of a patient, often on an experimental and/or compassionate basis’ [1]. This definition has been difficult to apply in practice as it was unclear to clinicians what extent of modification to an existing therapy was required to be considered innovative, what level of ‘proof’ of efficacy was required and that many medicines obtained via compassionate access programmes have significant evidence for use and therefore would not be considered innovative. Thus, although this detailed framework has been used in practice at GOSH for some years [13], their definition of innovative therapy has proven difficult to utilise in the Australian healthcare context due to differences in health system structure, pharmaceutical regulation and local medicines governance processes.

While the eight principles set out in the GOSH framework provide a useful and valid reference point, the limitations mentioned above prompted us to consider how they could be adapted for practical use in the contemporary Australian healthcare context.

## 2 Aim

The aims of this study were to develop and implement a working definition and ethical framework for use of innovative medicine in contemporary Australian paediatric practice, based on the framework developed at GOSH [1].

## 3 Methods

The study was an embedded mixed methods design, including a national Delphi study [14, 15] and a retrospective clinical audit of IPAs for non‐formulary items at Children's Health Queensland Hospital and Health Service based at the Queensland Children's Hospital, a quarternary paediatric health service in Brisbane, Australia.

### 3.1 Part A

Part A of the study involved a retrospective clinical audit of IPAs at the study site from 1 January to 31 December 2022. Data was extracted from the local IPA electronic database. Inclusion criteria were new applications recorded in the IPA database. Exclusion criteria included renewal applications, if the patient was over 18 years of age, or if the medication was already on the formulary (either Queensland Health List of Approved Medicines or the local Patient Group Approvals List) at the time of the IPA application.

Data collected included drug name and formulation, indication for use, age of patient at commencement of therapy, Australian Registration of Therapeutic Goods (ARTG) inclusion (drug and formulation) and registration with other international regulatory bodies with similar levels of quality control (European Medicines Agency in Europe, Food and Drug Administration in the United States of America and the Medicines and Healthcare Products Regulatory Agency in the United Kingdom).

A selection of these applications was deidentified and used as examples during Part A of the project to assist definition of an innovative medicine.

### 3.2 Part B

Delphi participants were recruited from expert paediatric medicines governance groups, including the National Paediatric Medicines Forum [10], Australian paediatric medication governance groups and paediatric ethics consultation services. Participants were invited via email to participate in the study and encouraged to share the survey with their teams to better capture breadth of opinions.

Round 1 presented the GOSH definition and framework [1, 13] along with examples of IPA applications at the study site identified during the clinical audit in Part A to aid deliberation and identification of themes.

Round 2 included examples of innovative medicines submitted by the survey group during Round 1. Responses to these examples assisted in drafting the definition.

Round 3 and 4 continued to refine the definition and framework, based on results from previous rounds. No medicine examples were used in these rounds.

Each question in the survey utilised a 7‐point Likert scale from strongly disagree to strongly agree. Free text space was provided for comments and feedback. Consensus was predefined as a minimum 80% of responses being 6 or higher in the 7 point scale. Four rounds were used to develop a consensus for definition and framework. Each round lasted 3 weeks.

Microsoft Forms was used to circulate the questions and collate responses. All responses were non‐identifiable to the project team.

### 3.3 Part C

Quantitative data from Part A was reviewed using the definition derived in Part B. IPA applications for medicines that were not on the ARTG were independently reviewed by three members of the research team to determine whether they were innovative using the innovative medicine definition developed in Part B.

A flowchart of the method is included in Figure 1.

**FIGURE 1:**
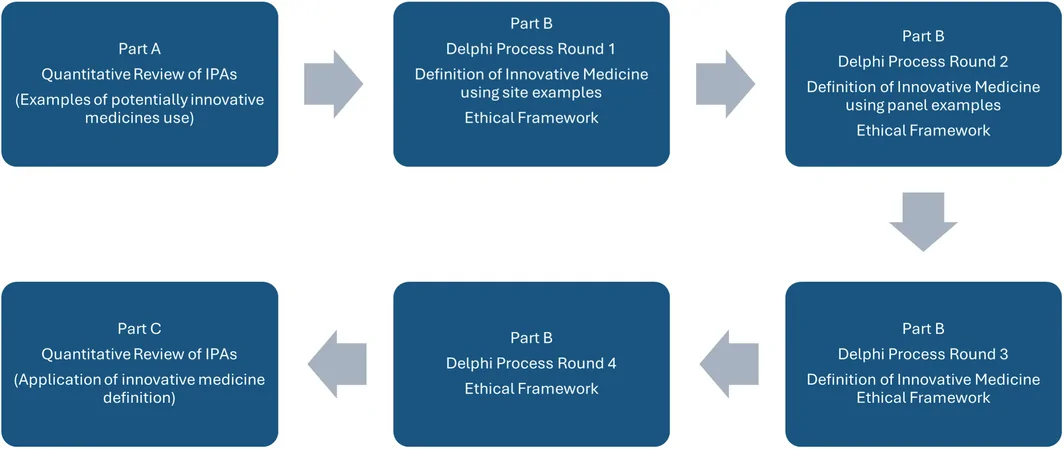
Method.

## 4 Ethics

All research was conducted in accordance with the National Statement on Ethical Conduct in Human Research (2007) [16]. This project was provided an ethics exemption as a quality assurance project by the Children's Health Queensland Hospital and Health Service Human Research Ethics committee (EX/2023/QCHQ/95378).

## 5 Results

### 5.1 Parts A—Quantitative Analysis of IPAs—Innovative Examples

A total of 2906 IPA applications were submitted during 2022. Of these, 1479 were excluded, mostly as they were renewal applications, with a small number excluded as the young person was over 18 years of age or the medication was on the hospital formulary. Of the 1447 included applications for analysis, 15 were selected for inclusion in Part B as examples of medicines use that may be considered innovative.

### 5.2 Part B—Delphi Process

A total of 18 individuals registered for the Delphi process. These included clinicians from Queensland, New South Wales, South Australia and Western Australia. Participants included pharmacists (11) and medical officers (7), with some dual identifying as researchers and ethicists; Round 1 also captured a response from a nurse who had not pre‐registered. Each round had between 10 and 16 respondents.

#### 5.2.1 Examples of Potential Innovative Uses of Medication

Round 1 used 15 examples from the study site experience, and 12 examples submitted from the survey participants were used in Round 2.

Factors that made a medicine MORE likely to be considered innovative included:

- Limited evidence to support the efficacy of use.
- Use outside of ARTG approved indication, especially if the indication is not approved by other international regulatory bodies
- Medicine used in a different formulation AND via a different route.
- Medicine itself is not approved by any other international regulatory agencies.

Factors that indicated the use was NOT innovative:

- Medicine used outside of approved age group (but same indication).
- Different formulation of a medicine approved for that indication (but the same route).
- Medicine not approved for use in Australia but approved for that indication by other regulatory bodies.

Results from the examples are available in the Supporting Information.

Consensus was reached for definition in Round 3 of the Delphi survey, with 90% of respondents answering 6 or higher. The updated definition is in Table 1.

**TABLE 1:** Definition of innovative medicine in paediatrics.

*An innovative medicine in paediatrics* is defined as any newly introduced medication (or new use for an existing medication) with limited published high‐level evidence of efficacy and safety for that indication, patient cohort, formulation and/or route of administration. High‐level evidence to support use may include approval by international regulatory agencies for the same or similar indications and route of administration. Use of a medication for an unapproved age group, in isolation of other factors, is not considered innovative. Consideration of a medication as innovative is dynamic as evidence evolves and applies to a specific moment in time.

#### 5.2.2 Ethical Framework

When considering the GOSH ethical framework [1], although the participants largely agreed with the eight principles, there were a number of themes that arose in applying the principles into practice, and suggestions for improvement to the framework:

- Assessment of routine or standard alternative therapies should be a precursor to the use of innovative therapies.
- Resource implications should be considered, including consideration of provision to future patients with similar conditions.
- The decision to treat with an innovative medicine should include more parties than just the treating team, including the patient and their family, and opinions from other experts or multidisciplinary governance groups.
- The value of a second opinion from experts external to the clinical team was emphasised.
- Coercion from the treating team must be prevented, while noting that coercion from the family to the treating team for the use of innovative therapies is an increasing concern.
- The importance of reporting results, preferably by publication but at a minimum reporting back on results to the governance group of the organisation (e.g., MTC). Potential use of a national register to build evidence for rare diseases was suggested.
- Timeliness of decision making, particularly when a patient's life is in danger, was reiterated, as was establishing clear outcome criteria to continue or stop therapy.
- The use of animal research was considered unlikely in contemporary practice, but other evidence such as genetic testing was increasingly valuable in identifying new potential therapies.
- There were differences of opinion on whether ethical evaluation should be undertaken before, after or integrated into clinical governance processes (such as IPAs).

A final list of nine principles to guide clinicians in the ethical evaluation of innovative medicines reached consensus in Round 4 (Table 2). Changes from the original framework were predominantly additional points of clarification as above, and an additional (ninth) principle specifying criteria for monitoring safety and outcomes established prior to commencement of therapy (see Table 2).

**TABLE 2:** Ethical framework for facilitating clinical governance evaluation of innovative medicines use in children.

There is a clear clinical need for this particular paediatric patient. All standard therapies have been trialled or considered not suitable for this patient. There is a realistic and reasonable scientific basis or biological plausibility (e.g., genetic testing) for what is proposed, which should be verified by an independent second opinion or review. There is a realistic expectation from the available evidence that the balance between benefit and potential harm or burden is in favour of treatment. There is consensus in the clinical team that what is proposed is in the patient's best interests and that there are no other reasonable or feasible or safer alternatives that might achieve the same result. Consensus could also include an independent opinion such as a Drug and Therapeutics Committee. The patient's family, and the patient if competent or able to understand, actually does understand that the therapy is experimental and what its possible risks, benefits and alternatives are. Information should be provided in an appropriate format and standard for the patient and family, and consent obtained and documented as per local governance processes. Allowing for the pressures produced by the patient's clinical condition, there should be no coercion or influence from the treating team, and the family should be free to withdraw at any time. The resources utilised by offering the treatment must be justified against likely success for that patient. Due consideration should be given to equity of access to resources for other patients in the same or similar circumstances. The results of the use should be reported, whatever the outcome. Criteria for monitoring for safety and outcomes must be established prior to commencement and an endpoint to treatment determined if outcome measures are not met.

### 5.3 Part C—Quantitative Analysis of IPAs

Of the 1447 IPAs that met inclusion criteria, 120 were medicines that were not on the ARTG, regardless of formulation. At time of review, six of these applications were considered innovative using the definition developed in Part B and included:

- Caffeine Citrate for myoclonus dystonia.
- Canakinumab for Hyper‐IgD syndrome mevalonate kinase deficiency.
- Evinacumab for homozygous familial hypercholesterolaemia (two cases).
- Fenfluramine for Lennox–Gastaut syndrome.
- Narsoplimab for treatment of transplant‐associated thrombotic microangiopathy (TA‐TMA).

Each of these examples was for use for indications that were not approved by any regulatory body or high level evidence available for use at that time.

## 6 Discussion

This study was able to develop and reach consensus on both a definition for innovative medicines in paediatrics and an ethical framework suitable for use in clinical governance evaluation in Australian settings.

Use of real world and recent examples of potentially innovative use of medicines allowed clarification of the definition to ensure applicability for routine use. Factors that contributed to a medicine being considered innovative included lack of rigorous Australian and international evaluation. The panel's conclusion that using a medication for the same approved indication but outside the approved age range did not indicate that the use was innovative echoed ongoing concerns regarding equitable access to medicines in paediatric patients, particularly in Australia due to a relatively smaller population [3, 6, 17]. Another key point was the dynamic nature of medicine utilisation, particularly in paediatrics; as any designation of a medication as innovative applies at a single point of time and evolves as new evidence arises. This was reflected in one of the examples of the use of fenfluramine for Lennox Gastaut Syndrome, which was innovative at the time that the medicine was first used (in 2022) but would now not be considered innovative due to the accumulated evidence over subsequent years.

As expected, the fundamental elements of the GOSH framework remain relevant, given they reflect the core ethical principles of autonomy, justice, beneficence and non‐maleficence [18]. Our framework provides additional context regarding balance of benefit and burden or harm of intervention; ensuring equitable access (incorporating aspects of distributive justice); the importance of sharing results of outcomes and clarity on criteria for monitoring safety and outcomes. The use of innovative medicines in individual paediatric patients has parallels with research ethics, and our framework is consistent with the National Statement on Ethical Conduct in Human Research [16]. Our framework is consistent with and provides additional guidance to support the new guiding principle of ‘MTCs implement transparent and ethically robust processes’ in the updated 2025 CATAG MTC guidance document [11].

## 7 Limitations

Not all feedback from the expert panel was incorporated into the ethical framework. Particularly, references to specific local governance processes were consciously avoided as these are organisation specific rather than an approach that could be implemented broadly, and there was no consensus from the comments provided. What was clear from participants was that explicit inclusion of clinical ethics into clinical governance processes was desired, whether that was with an ethicist as part of the Medicines and Therapeutics Committee and/or by routine use of an ethical framework such as this. During survey recruitment, the inclusion criteria was for people currently working in paediatrics. This was to ensure the challenges and considerations unique to paediatrics were addressed in the framework, but did introduce an inherent but intentional bias. This does limit the applicability of results to paediatrics; however, as no input from clinicians working in adult services was included.

The expert panel did not include members from all Australian states and territories, despite wide invitations. Nevertheless, the states that participated reflected the range of processes across Australian healthcare settings and ensure applicability across Australia.

Initial reservations of additional administrative burden into clinical governance steps were allayed by the small number of innovative medicine applications identified per year at the study site. Since completion of the framework, it has been integrated into clinical governance evaluation of innovative medicine applications by the local MAC, with positive feedback from the committee members.

## 8 Conclusion

This study has affirmed the importance of integrating ethical evaluation into clinical governance processes as an essential component of paediatric clinical care, particularly in the use of innovative medicines. The criteria included in the framework described in this paper facilitate this to occur in a consistent and structured manner.

## Author Contributions

**Sonya Stacey:** conceptualisation (lead), methodology (lead), supervision (lead), writing – draft preparation (lead), writing – review and editing (lead). **Jamie Lam:** conceptualisation (supporting), data curation and formal analysis (lead), methodology (supporting), writing – draft preparation (supporting), writing – review and editing (supporting). **Fiona Gregory:** conceptualisation (supporting), data curation and formal analysis (supporting), methodology (supporting), supervision (supporting), writing – review and editing (supporting). **Shakira Wild:** formal analysis (supporting), validation (lead). **Steven McTaggart:** methodology (supporting), writing – review and editing (supporting). **Helen Irving:** conceptualisation (supporting), methodology (supporting), supervision (supporting), writing – review and editing (supporting).

## Funding

The authors have nothing to report.

## Ethics Statement

This project was provided an ethics exemption as a quality assurance project by the Children's Health Queensland Hospital and Health Service Human Research Ethics committee (EX/2023/QCHQ/95378).

## Consent

The authors have nothing to report.

## Conflicts of Interest

The authors declare no conflicts of interest.

## Supporting information


**Supporting Information: S1.** Examples of innovative medicines used in Rounds 1 and 2 of the Delphi Process (Part B).

## Data Availability

The data that support the findings of this study are available on request from the corresponding author. The data are not publicly available due to privacy or ethical restrictions.
