# Supplementary material for: A Framework for Ethical and Clinical Governance Evaluation of Innovative Medicines in Children: A Mixed Methods Study
Source: J Paediatr Child Health. 2026 Jul 25;62(9):1693–9. doi: 10.1111/jpc.70501 (PMC13617224; doi:10.1111/jpc.70501)
Supplement: Supplementary file 1 [file JPC-62-1693-s001.docx]

## Supplementary Information

Table below includes the examples of innovative medicines used in Rounds 1 and 2 of the Delphi Process (Part B). Responses ranged from 1 (strongly disagree) to 7 (strongly agree) that the medication is innovative. Lower mean scores indicate that the medicine and use is NOT innovative; higher mean scores indicate that the medicine and use IS considered innovative.

| Example | Themes | Mean Response |
| --- | --- | --- |
| Cannabidiol, in a formulation manufactured by Tilray being used as adjunctive therapy to treat epilepsy in children. The Epidyolex brand of cannabidiol is the only currently approved formulation by the TGA as adjunct therapy for seizures in Lennox-Gastaut syndrome or Dravet syndrome. The Tilray product is not ARTG-listed but is approved by the Canadian regulatory authority, the Health Product and Food Branch. | Formulation not registered locally but being used as per registered indications. RCTs available to support this use. | 3.25 |
| Escitalopram, which is ARTG listed for treatment of major depression in patients 18 years and over, being used to treat major depression in paediatric patients. | TGA approved, approved for that indication but not age group. RCTs support safety of use in age group. | 3.60 |
| Fenfluramine, a drug approved by the EMA and FDA but not the TGA, being used as indicated for intractable seizures associated with Dravet syndrome in children. | Internationally approved for this indication, no local approval. Randomised controlled trials (RCTs) and case studies to support use. | 3.69 |
| Dupilumab, being used for severe atopic dermatitis as indicated but for patients under 6 years old, who are under the TGA specified age range of 6 years and over. It has been approved for patients from 6 months to 5 years years as well by the FDA. | Registered medication used as per approved condition but for patients outside of approved age range. RCT and case reports to support this use, | 3.81 |
| Ondansetron, which is only TGA approved for treating postoperative nausea and chemotherapy related nausea and vomiting, being used to treat acute gastroenteritis in paediatrics. This specific indication is not approved internationally by the EMA or FDA either. | Locally registered formulation and medication used for unapproved an indication. RCTs and systematic reviews have studied this use in paediatric populations. | 3.94 |
| Fluoxetine, which is ARTG listed for treatment of major depression in patients 18 years and over, being used to treat major depression in paediatric patients. | TGA approved drug, approved indication, unapproved age group. RCTs support safety and efficacy of use. | 3.94 |
| Drug X, an ARTG listed medicine with a narrow therapeutic index, being used with Bayesian forecasting to minimise toxicity while maintaining therapeutic concentrations. Doses are normally otherwise based purely on patient weight. | Registered medication used as per indication but with novel dosing and monitoring regimens. | 4.13 |
| Zoledronate, an ARTG-listed drug, being used as an adjuvant therapy for hypercalcaemia in a cancer care protocol to treat Aneurysmal Bone Cyst. It is approved by the FDA for this indication in adults but not by the EMA. | Locally registered for a different indication. This indication is approved internationally and has a case report to support its use. | 4.38 |
| Tofacitinib, normally used to treat alopecia areata universalis which it is indicated for, but compounded into a topical form which is not approved. | Registered medication for an approved indication but in an unregistered formulation. Case reports have studied this use with unclear results. | 4.38 |
| Iloprost, which is ARTG listed for treatment of primary or secondary pulmonary hypertension in adults, being used to treat pulmonary hypertension refractory to sildenafil and nitric oxide in a newborn. | TGA approved, indication approved. Unapproved age group, case reports available. | 4.38 |
| Sodium bicarbonate injection, which is ARTG listed for treatment of metabolic acidosis in patients 2 years and over, being used as an alkalising agent to reduce pain from lidocaine injections in children 2 years and over. | TGA approved, unapproved indication, unapproved age group. RCTs and systematic reviews available. | 4.38 |
| Sotrovimab, ARTG listed for treatment of COVID-19 for patients 12 and older who do not require ventilation but are at high risk of progressing to severe disease, being used to treat COVID-19 in immunocompromised children under 1 year of age. | TGA approved, unapproved for indication or age group. No published evidence available. | 4.60 |
| Elapegadamese, being used to treat severe combined immunodeficiency (SCID), which is not approved in Australia but is approved overseas by the FDA only. | Medication not registered locally but being used as per international approval. Clinical trial used for regulatory approval not yet formally published. | 4.69 |
| Infliximab, an ARTG-listed drug, being used under a compassionate access program to investigate its use, specifically high dose infliximab for Inflammatory Bowel Disease (higher than the PBS approved dosing). | Locally registered for this indication but not this dose. Systematic reviews and RCTs to support this use. | 4.75 |
| Budesonide nebuliser solution being used orally (topically in the throat, not swallowed), by mixing into a more viscous preparation, to treat eosinophilic oesophagitis. | Registered formulation used for an unapproved condition via a different route of administration. RCT and case reports that support this use. | 4.75 |
| Trametinib, a drug listed on the ARTG for treatment of melanoma, being used as an oral solution, which is not approved by the TGA, for the unapproved indication of plexiform neurofibroma. This medicine is available for free of charge as part of Novartis's medicines access program. The oral solution has been approved by the EMA. | Formulation not registered locally and used for an unapproved indication. Both formulation and indication are have international approval. Case reports to support this use. | 5 |
| Artesunate, which has been approved by the FDA and EMA for treatment of severe malaria in adult and paediatric patients, being used for malaria prophylaxis for a premature neonate with a malaria positive mother. | International approval, not locally approved. Indication unapproved, age group unapproved. Safety data from case reports, nil for efficacy. | 5.10 |
| Pitolisant, a drug approved by the EMA and FDA for excessive daytime sleepiness in adult patients with narcolepsy, being used to treat narcolepsy a in paediatric patient with Prader-Willi syndrome. | Not approved by TGA, internationally approved. Approved indication (internationally), unapproved age group. RCT and case study evidence available. | 5.10 |
| Ataluren, which has only been approved by the EMA for Duchenne Muscular Dystrophy (DMD), after failing FDA registration due to being unable to prove sufficient efficacy data, being used to treat DMD where there are no other alternative disease modifying agent available. | Unregistered medication used as per international approval to treat a rare condition. RCTs have examined this use. | 5.19 |
| Ketamine wafers, as the product Wafermine which is not approved by the FDA or EMA, being used to treat breakthrough pain in paediatric patients. | Approved medicine, unapproved formulation for different route of administration. Unapproved indication. Approved for age group with different formulation/route. Clinical trials supporting use in adult populations. | 5.20 |
| Tetrahydrocannabinoid and Cannabidiol combination oil used to treat dystonia in palliative care.  Cannabidiol alone is approved by the TGA as Epidyolex to treat seizures in Lennox-Gastaut syndrome and Dravet syndrome. It is not approved for this indication by the FDA or EMA. | Formulation not registered locally and used for an unapproved indication. Case report available which has investigated another drug from the same class for this use. | 5.25 |
| The combination product Trikafta (Elexacaftor/Tezacaftor/Ivacaftor), which is ARTG listed for treatment of cystic fibrosis (CF) secondary to f508del CFTR gene mutation in patients 6 years and over, being used to treat respiratory conditions such as COPD which may be related to an acquired CFTR gene dysfunction in patients who do not have CF. | TGA approved, unapproved indication, unapproved age group. Limited evidence for this use. | 5.80 |
| Paxalisib being used as per clinical trial protocol for diffuse intrinsic pontine glioma (DIPG), where it is not approved anywhere internationally. | Unregistered medication, locally or internationally, used in clinical trials. RCTs available for this use in adult populations. | 6.00 |
| Ruxolitinib, ARTG listed for treatment of acute host vs graft disease in patients 12 years and over, being used to treat bronchiolitis obliterans in an 8 year old patient. | TGA approved, unapproved indication, unapproved age group, no available evidence. | 6.10 |
| A drug not yet approved by any regulatory authorities, currently undergoing investigation as part of a clinical trial.  Use would be under compassionate access from the manufacturer, for the indication currently being investigated but where the child is not eligible for enrolment in the trial. | Unregistered medication on clinical trial used per protocol but not as part of the trial. | 6.19 |
| Tazemetostat, a drug not ARTG listed but approved by the FDA for treatment of epithelioid sarcoma in patients 16 years and above and relapsed or refractory follicular lymphoma in adults, being recommended to treat an atypical tumour in a patient under 12 as per tumour testing. | TGA approved product, unapproved indication, unapproved age group. No published evidence for this use | 6.20 |
| Tiratricol, a drug not ARTG-listed and unapproved by the EMA and FDA, being used to treat Allan-Herndon-Dudley Syndrome (an orphan disease). | Unapproved by TGA, unapproved indication, unapproved age group. No published evidence, ongoing clinical trials. | 6.20 |
